# Vicarious Menstruation

**Published:** 1886-07

**Authors:** 


### Vicarious Menstruation.

Puech has collected 200 cases of vicarious menstruation, in all which a sanguinolent mucus was secreted in the genitals during the catamenial periods. The hemorrhage came from—

| | |
|---|---|
| The hair follicles, . . . | 6 cases. |
| The ear, . . . . . | 6 " |
| The lachrymal tracts, . . . | 10 " |
| The nose, . . . . . | 18 " |
| The gums, . . . . | 10 " |
| The cheeks, . . . . | 3 " |
| The mouth, . . . . | 4 " |
| The bronchi, . . . . | 24 " |
| The stomach, . . . . | 32 " |
| The breast, . . . . | 25 " |
| The axilla, . . . . | 10 " |
| The umbilicus, . . . . | 5 " |
| The bladder, . . . . | 8 " |
| The intestine, . . . . | 10 " |
| The hands, . . . . | 7 " |
| Other regions, . . . . | 21 " |

---

In an article published in the *Revista Med. Quirurg. de Buenos Ayres*, Dr. J. Franceschi states that no case of rabies has been observed in that province during the past fifteen years, although the proportion of dogs kept there is larger than in any European country. He believes that rabies may be produced idiopathically in dogs by feeding them on vegetable food exclusively. In Europe, meat is scarce, and hydrophobia correspondingly common, but in Argentine Republic, meat is the common food of dogs, and hydrophobia is unknown.

